# *Streptococcus agalactiae* disrupts P-glycoprotein function in brain endothelial cells

**DOI:** 10.1186/s12987-019-0146-5

**Published:** 2019-08-22

**Authors:** Brandon J. Kim, Maura A. McDonagh, Liwen Deng, Benjamin D. Gastfriend, Alexandra Schubert-Unkmeir, Kelly S. Doran, Eric V. Shusta

**Affiliations:** 1 Department of Chemical and Biological Engineering, University of Wisconsin, Madison, WI USA; 2 Department of Immunology and Microbiology, University of Colorado School of Medicine, Aurora, CO USA; 3 Department of Hygiene and Microbiology, University of Würzburg, Joseph Schneider Strasse 2/E1, 97080 Würzburg, Germany

**Keywords:** Group B *Streptococcus*, *Streptococcus agalactiae*, Brain endothelial cells, *P*-glycoprotein, Efflux transport, Meningitis, Stem cells, P-gp

## Abstract

Bacterial meningitis is a serious life threatening infection of the CNS. To cause meningitis, blood–borne bacteria need to interact with and penetrate brain endothelial cells (BECs) that comprise the blood–brain barrier. BECs help maintain brain homeostasis and they possess an array of efflux transporters, such as *P*-glycoprotein (P-gp), that function to efflux potentially harmful compounds from the CNS back into the circulation. Oftentimes, efflux also serves to limit the brain uptake of therapeutic drugs, representing a major hurdle for CNS drug delivery. During meningitis, BEC barrier integrity is compromised; however, little is known about efflux transport perturbations during infection. Thus, understanding the impact of bacterial infection on P-gp function would be important for potential routes of therapeutic intervention. To this end, the meningeal bacterial pathogen, *Streptococcus agalactiae,* was found to inhibit P-gp activity in human induced pluripotent stem cell-derived BECs, and live bacteria were required for the observed inhibition. This observation was correlated to decreased P-gp expression both in vitro and during infection in vivo using a mouse model of bacterial meningitis. Given the impact of bacterial interactions on P-gp function, it will be important to incorporate these findings into analyses of drug delivery paradigms for bacterial infections of the CNS.

**Electronic supplementary material:**

The online version of this article (10.1186/s12987-019-0146-5) contains supplementary material, which is available to authorized users.

## Introduction

The blood–brain barrier (BBB) and other brain barriers such as the meningeal blood-cerebrospinal fluid barrier are comprised of highly specialized brain endothelial cells (BECs) that promote proper brain function by separating the circulation from the central nervous system (CNS) [1–3]. BECs possess unique phenotypes that include the presence of specialized efflux transporters, complex tight junctions, and low rates of endocytosis [1–3]. Together, these properties maintain brain homeostasis and help to prevent the entry of pathogens and toxins into the CNS. P-glycoprotein (P-gp) is a major efflux transporter expressed in BECs that is able to efflux a wide variety of lipophilic molecules back into the bloodstream and because of this, can regulate the CNS accumulation of drugs [4–9]. Much work has been conducted to understand and modulate P-gp function at the BBB in order to enhance drugs access to the brain [4–9]. Moreover, the effects of disease conditions on P-gp function are increasingly being explored [10–13].

Bacterial meningitis is a serious infection of the CNS that occurs when blood-borne bacteria are able to breach BECs and cause inflammation [14–17]. Certain pathogens possess virulence factors that promote BEC interaction, and previous studies have characterized the molecular interactions that result in penetration of brain endothelium [14–17]. *Streptococcus agalactiae*, also known as Group B *Streptococcus* (GBS), is a Gram-positive bacterium that is the leading cause of neonatal meningitis [18]. GBS disruption of the BECs and other brain barriers during meningitis has been documented, and it has been shown that tight junctions are disrupted and endocytosis pathways altered [19, 20]. However, little is known about the effect of bacterial infection on P-gp function. Here, we show that bacterial infection can alter P-gp function in BECs, suggesting another mechanism by which bacterial pathogens contribute to BEC dysfunction.

## Results

### Group B Streptococcus infection inhibits BEC P-gp function

**Fig. 1:**
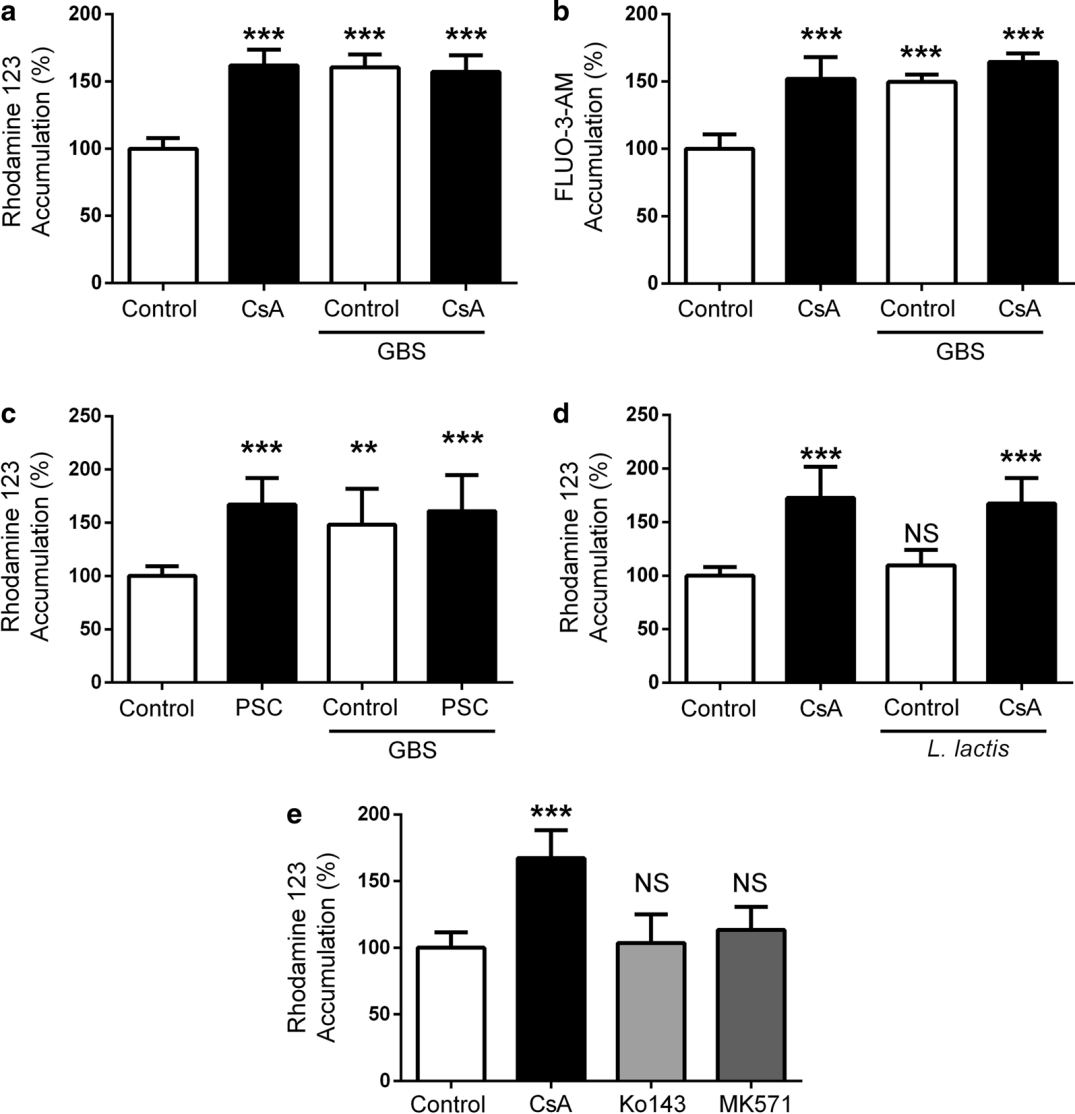
GBS effects on iPSC-derived BEC P-gp function. **a** BECs were either uninfected (control) or infected with GBS (MOI = 10) for 5 h. After infection, P-gp activity was measured by observing R123 accumulation with or without CsA inhibition. **b** P-gp activity measured using FLUO-3-AM as a P-gp substrate. Experimental groups and infection times are as described in (**a**). **c** Just as in (**a**), BECs were either left uninfected (control) or infected with GBS (MOI = 10) for 5 h. P-gp activity was measured by observing R123 accumulation with or without PSC-833 (PSC) as the inhibitor **d**. P-gp activity assay monitoring R123 accumulation as in (**a**) for BECs incubated with non-pathogenic *L. lactis* (MOI = 10) for 5 h. **e** BECs tested for R123 accumulation after treatment with the P-gp inhibitor CsA, BCRP inhibitor Ko143, and MRP family inhibitor MK571. Experiments were performed at least in triplicate on three independent differentiations (**a**–**c**, **e**, n = 9), or in triplicate on two independent differentiations (**d**, n = 6). All data are presented, and expressed as % of uninfected control BEC accumulation for all experiments, and bars represent mean ± SD. **p < 0.01, ***p < 0.001, NS p > 0.05, versus uninfected control; ANOVA followed by Dunnett’s multiple comparisons test

We first sought to determine if P-gp function was altered during infection. To do so, we utilized induced pluripotent stem cell (iPSC)-derived BEC-like cells that have been shown to possess P-gp activity [21–23]. iPSC-derived BECs were differentiated and express expected endothelial markers as previously described (Additional file 1: Figure S1A-F) [21–23]. Using a substrate accumulation assay and consistent with prior observations, BECs treated with the P-gp inhibitor Cyclosporine A (CsA) accumulated more of the P-gp substrate Rhodamine 123 (R123) than non-CsA-treated cells, indicating that P-gp is active in these BECs. Following GBS infection, we observed a significant increase of R123 accumulation in BECs when compared to uninfected BECs, to levels matching those with CsA inhibition (Fig. 1a). Addition of CsA to the infected condition made no impact on accumulation, and the combined data suggest P-gp function is diminished during GBS infection. To determine if the observation was substrate specific, similar experiments were performed with a different P-gp substrate, FLUO-3-AM, and a similar increase in substrate accumulation in GBS-infected BECs was observed (Fig. 1b). In addition, inhibition of P-gp with the second generation, more specific inhibitor PSC-833 [6] yielded similar results to CsA inhibition (Fig. 1c). To determine if this impact on P-gp function is specific to meningeal pathogens, P-gp function was assayed following incubation with a genetically similar non-pathogenic bacterium, *Lactococcus lactis*. In contrast to GBS effects, *L. lactis* did not inhibit P-gp function in an R123 accumulation assay (Fig. 1d). We and others have previously shown that inhibition of BCRP or MRP family proteins with Ko143 or MK571 respectively, in iPSC-BECs resulted in functional inhibition of those transporters [21, 22, 24–29]. Inhibition of BCRP or MRPs using Ko143 or MK571, did not result in an increase in R123 accumulation (Fig. 1e). This suggests that R123 efflux, in our model, is primarily mediated by P-gp and that accumulation can be increased by CsA. Taken together, these observations suggest that GBS infection results in reduced P-gp function in BECs as measured by substrate accumulation.

### Group B Streptococcus infection inhibits function in P-gp overexpressing cells

**Fig. 2:**
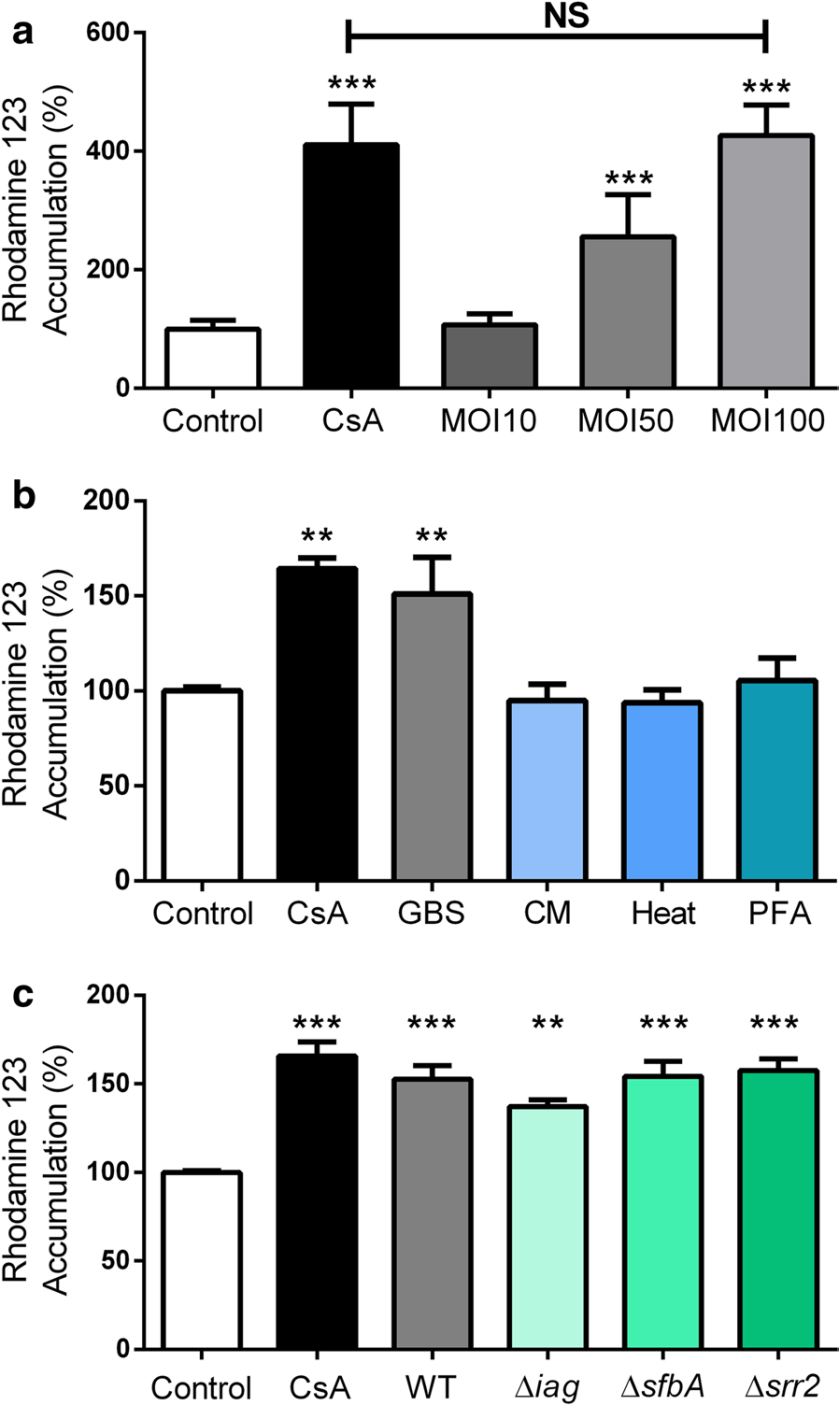
Bacterial interactions regulating P-gp inhibition. **a** Effects of GBS infection on R123 accumulation in an MDCK cell line that overexpresses human *MDR1*. Multiplicity of infection (MOI) was varied and after 5 h of infection, P-gp function was compared to uninfected cells and CsA treated cells. **b** Effects of live GBS, GBS-conditioned medium (CM) and heat-treated non-viable (Heat) or paraformaldehyde-fixed (PFA) GBS on R123 accumulation in iPSC-derived BECs after 5 h of treatment. **c** iPSC-derived BECs were infected with GBS mutants or wild-type (WT) (MOI = 10) for 5 h, and R123 accumulation compared to uninfected controls. Experiments were performed in triplicate on three independent biological replicates (**a**) or differentiations (**b**, **c**). All raw data are presented (n = 9), and are expressed as % of uninfected control BEC accumulation. All experiments and bars represent mean ± SD. **p < 0.01, ***p < 0.001, NS p > 0.05, versus uninfected control unless otherwise specified; ANOVA followed by Dunnett’s multiple comparisons test

To ensure that the observed decrease in P-gp was not a function of the iPSC origin of BECs, we also examined P-gp function in a human P-gp-overexpressing Madin-Darby canine kidney (MDCK) cell line [30]. As a result of the overexpression of human P-gp, there is a greater relative increase of R123 accumulation upon CsA inhibition (Fig. 2a). Despite this increase in P-gp activity, GBS-mediated P-gp inhibition could still be observed with complete inhibition of P-gp activity at high multiplicity of infection (MOI) (Fig. 2a). These data suggest that P-gp inhibition is related to the balance of P-gp expression and level of bacterial interaction, and that GBS-mediated P-gp inhibition may not be specific to BECs.

### Live GBS is required for inhibition of P-gp activity

We performed accumulation experiments to examine if a secreted factor was responsible for inhibiting P-gp activity. Exposure of BECs to GBS-conditioned medium had no influence on P-gp function, indicating a requirement for GBS-BEC interactions (Fig. 2b). Next, we investigated if bacterial components, but not living bacteria, could generate P-gp inhibition. Interaction of heat-treated, nonviable GBS or paraformaldehyde-fixed GBS with BECs also did not affect P-gp function (Fig. 2b). These results suggest that interaction with live GBS is required for P-gp inhibition as secreted GBS components or nonviable forms of GBS were insufficient to decrease P-gp function. Given this requirement, and the fact that previous work has identified various GBS virulence factors that contribute to overall bacterial-BEC interactions, we next tested a number of bacterial mutants identified to contribute to bacterial interaction with BECs. We examined GBS mutants lacking surface expressed adhesins SfbA and Srr2, and an invasion associated gene (*iagA*) that functions to properly anchor bacterial lipoteichoic acid, to see if any of the virulence factors contributed to P-gp inhibition during infection [31–34]. BECs infected by each of the mutants exhibit similar P-gp substrate accumulation to BECs infected by the wild-type (WT) GBS, and all show significantly increased accumulation over uninfected controls (Fig. 2c). Taken together, these data suggest that live GBS is required for P-gp inhibition, and SfbA, Srr2 or iagA alone does not mediate that P-gp inhibition.

### P-gp expression and abundance decreases during GBS infection

**Fig. 3:**
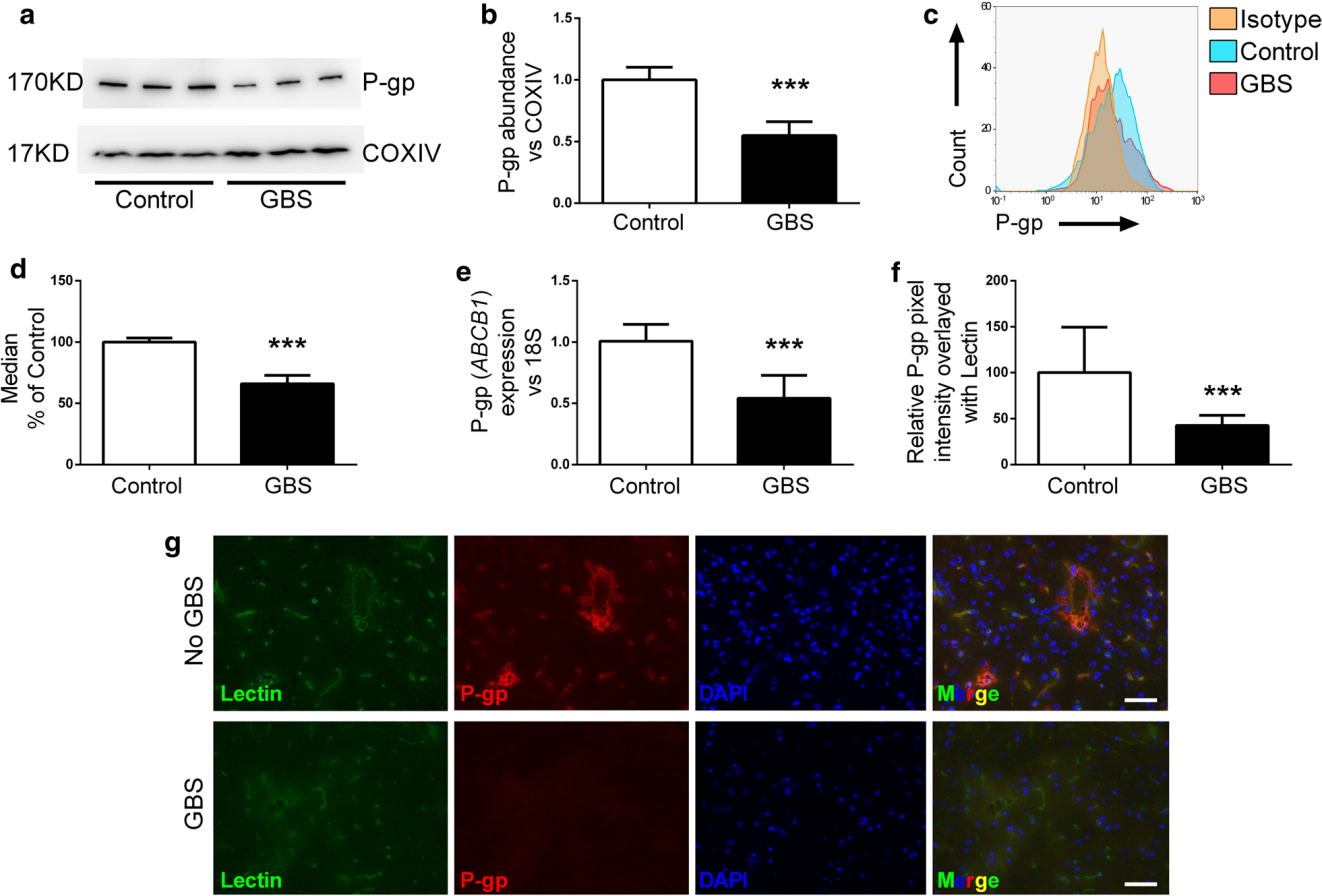
Effects of GBS infection on P-gp expression in BECs. **a** BECs were infected with GBS (MOI = 10) for 5 h, and protein lysates were collected. Western blot analysis was performed on lysates using COXIV as a loading control, representative blot shown. **b** Densitometry was conducted to determine fold change in P-gp expression using FIJI ImageJ software after normalization to respective COXIV bands on all experiments performed in triplicate on three independent differentiations (n = 9). **c** Representative flow cytometry histogram of BECs stained for total P-gp after 5 h of GBS infection (MOI = 10) compared with uninfected BECs (Control). **d** P-gp abundance is plotted as the median fluorescence intensity generated by flow cytometry normalized to uninfected control on all experiments performed at least in duplicate on three independent differentiations (n = 7). **e** qPCR performed for *ABCB1* (P-gp) and normalized to 18S rRNA for BECs with or without GBS infection at an MOI of 10 for 5 h. Data presented are from three experiments performed in triplicate. **f** Quantitation of relative P-gp pixel intensity overlayed with vascular lectin staining in mouse brains. Control mice (n = 5) with 12 FOV had 481 lectin positive vessels analyzed, GBS mice (n = 6) with 12 FOV had 544 lectin positive vessels analyzed. **g** Representative images of mouse brains stained for lectin (green), P-gp (red), and DAPI (blue) after GBS infection. Control mouse with no GBS in the brain (top), and mouse with GBS in the brain (bottom). Scale bar represents 50 μm. Data represent mean ± SD. ***p < 0.001; Student’s *t* test

To examine whether the decrease in P-gp activity correlated with a decrease in P-gp abundance, western blot and flow cytometry were used to quantify P-gp expression. BECs were infected with GBS, and western blot analysis was conducted on BEC lysates. We observed that overall P-gp abundance decreased during GBS infection (Fig. 3a, b). These results were confirmed by flow cytometry for P-gp where a decrease in P-gp expression was measured (Fig. 3c, d). RT-qPCR conducted on lysates further demonstrated that P-gp-encoding *ABCB1* expression was decreased upon GBS infection (Fig. 3e). To determine if this decrease was observed in vivo, we employed our murine model of GBS meningitis [19, 33, 35]. We observed that during GBS infection mice exhibited less P-gp immunolabeling that co-localized with endothelial cells (Fig. 3f, g, Additional file 2: Figure S2). Taken together, these data suggest that the disruption in P-gp activity in BECs after GBS infection may be due to a decrease in P-gp abundance.

## Discussion

Previous work has shown that GBS can interact with BECs resulting in tight junction disruption, an innate immune response, and alteration of other cellular processes [14, 19, 20, 36]. Here we observe for the first time that GBS is also able to disrupt BEC P-gp efflux transport. Using iPSC-derived BECs, an *MDR1* (P-gp) overexpressing MDCK line, and a mouse model of GBS infection we have established that P-gp function appears to be diminished during GBS infection.

The impact of GBS on P-gp was independent of substrate given the increased accumulation of two different substrates, R123 and FLUO-3-AM (Fig. 1a, b). Previously, we and others have demonstrated that iPSC-BECs possess functional BCRP and MRP that can be inhibited by Ko143 or MK571 respectively [21, 22, 24–29]. Inhibition of BCRP or MRP family proteins did not result in a significant increase in R123 accumulation suggesting that the CsA- induced increase in R123 accumulation is mediated by P-gp in the iPSC-BEC model (Fig. 1e). The more specific second-generation P-gp inhibitor PSC-833 (Valspodar) [6], showed a similar impact on R123 accumulation (Fig. 1c). In addition, the effects appear to be dependent on what bacterium is interacting with the BECs. For instance, while GBS affected P-gp function, another Gram-positive bacterium, *L. lactis*, that is non-pathogenic, did not impact P-gp function. More work is required to identify the specific bacterial factor in GBS responsible for the loss of P-gp function during infection, as several known GBS invasion mutants did not affect the observed P-gp inhibition (Fig. 2c). However, our data suggest that direct live bacterial challenge is required since neither conditioned medium nor nonviable bacteria resulted in the same functional inhibition of P-gp. Meanwhile, direct interaction of live GBS on a non-BEC cell line overexpressing P-gp was also sufficient for loss of function indicating that the effects may not be BEC-specific. Moreover in BECs, the decrease in P-gp function accompanied a decrease in P-gp expression.

P-gp function in BECs impacting neurological disorders such as Alzheimer’s disease has been examined, where decreased function is associated with more Aβ deposition in the brain [10, 12]. Interestingly, while modeling Huntington’s disease in iPSC-derived BECs, it was observed that P-gp function was decreased but expression was increased [11]. P-gp function is also altered in various inflammatory states. Isolation and treatment of primary BECs from guinea pigs with pro-inflammatory cytokines led to a decrease in P-gp function and expression similar to our observations [37]. In vivo, inflammatory molecules such as lipopolysaccharide (LPS) and cytokines have also been shown to decrease P-gp function while increasing P-gp expression in a murine model [38]. However in rats, LPS treatment resulted in both lower expression and function of P-gp in the brain, suggesting model and/or species dependent differences [39]. This disjointed observation between expression levels and function is further confirmed in other P-gp expressing tissues that have been challenged with LPS or bacteria with varying expression levels after treatment [40–45]. Further work will be required to determine mechanisms by which GBS infection diminishes P-gp function. We observed decreased expression of P-gp in BECs on both transcript and protein levels as a result of GBS infection (Fig. 3a–e). However, in the MDCK-MDR1 cells, human P-gp is overexpressed and is not under the control of endogenous P-gp gene regulatory systems [30], and yet we still observed disruption of P-gp function (Fig. 2a). These data then suggest that in addition to the decrease in expression, a bacterial factor may also directly interact with and contribute to the inhibition of P-gp.

In barrier forming intestinal epithelial cells, *Listeria monocytogenes* and *Salmonella enterica* serovar *Typhimurium* have been shown to disrupt P-gp function [46, 47]. In the case of *Listeria monocytogenes*, P-gp acted as protective factor to invasive infection, and Listerial proteins inhibited normal P-gp function by acting as a competitive substrate [47]. Additionally, *S. typhimurium* infection resulted in R123 accumulation into epithelial monolayers [46]. GBS is carried in up to 30% of healthy individuals in the gastrointestinal tract and could potentially be interacting with similar P-gp expressing epithelial cells in a colonization state [14, 48]. Invasive GBS late-onset disease in newborns manifesting in sepsis and meningitis up to 3 months postnatally coincides with increasing P-gp expression in the brain that reaches maximum levels between 3 and 6 months of life [49, 50]. It is possible that functional P-gp may possess BEC barrier protecting functions, such as in the case with *L. monocytogenes* in the gut [47], and may contribute to the decrease in invasive GBS disease after 3 months of life. Here we demonstrate that GBS can disrupt P-gp function in BECs, however further investigation is required to understand how inhibition might impact BEC barrier function and bacterial invasion.

Our study suggests that a generalized response to bacteria, such as through Toll-like receptor agonists, may not be sufficient to decrease P-gp function since non-pathogenic live bacteria, and nonviable GBS do not decrease P-gp function (Fig. 2b). Future work will be required to determine the precise mechanism of P-gp dysfunction during infection, signaling pathways involved with P-gp downregulation, and impacts other transporters in BECs. Finally, these findings suggest that P-gp inhibition should be taken into consideration when exploring therapeutic strategies for bacterial infection of the CNS.

## Materials and methods

### Bacterial strains and cell lines used

Induced pluripotent stem cell (iPSC) line IMR-90-C4 (WiCell) was maintained per previous reports and grown on Matrigel (WiCell) coated plates (Corning) in mTeSR1 medium (WiCell) changed daily. IMR-90 iPSCs were passaged twice a week as needed [21–23, 26, 36, 51]. MDCK-MDR1 [52] (ATCC) cells were maintained on tissue culture treated 25 cm^2^ flasks in DMEM (Life Technologies) + 10% Fetal Bovine Serum (WiCell). Group B *Streptococcus* (GBS, *Streptococcus agalactiae*) hypervirulent clinical isolate COH1 (serotype III, multilocus sequence type 17) strain was used [53]. Previously described COH1 mutants *Δiag* [33], *Δsrr2* [54, 55], and *ΔsfbA* [34] were employed for mutant analysis. All GBS strains were grown in static Todd-Hewitt broth (THB) at 37 °C. *Lactococcus lactis* was grown in M17 medium at 30 °C in static culture as previously described [56]. *L. lactis* was prepared exactly like GBS as mentioned above.

### Brain endothelial cell differentiation

iPSC-derived brain microvascular endothelial cells (BECs) were differentiated as previously described [21–23, 26, 36, 51]. To ensure the quality of the cultures, mycoplasma testing was conducted periodically through services from WiCell, and PCR based kit (PanReac AppliChem). Briefly, single cell suspension of iPSCs were seeded at a density of 10,000/cm^2^ onto Matrigel (WiCell) cell culture plates or flasks (Corning) and expanded for 3 days in mTeSR1 changing media daily. Initiation of differentiation was conducted by exchanging to unconditioned media (UM; DMEM-F12 base medium [Life Technologies], 20% Knockout serum replacement [Life Technologies], 1% minimal essential medium-nonessential amino acids [Life Technologies], 0.5% Glutamax [Life Technologies], and 0.07% beta-mercaptoethanol [Sigma]), for 6 days changing media daily. After the 6 days, media was then changed to EC medium (human endothelial cell serum-free media [Life Technologies], 1% platelet-poor plasma derived serum [Fisher], 500 ng/ml basic fibroblast growth factor, and 10 μM all trans-retinoic acid [Sigma]) for 2 days. Finally, differentiated BECs were purified onto collagen IV (Sigma) and Fibronectin (Sigma) coated plates and transwells inserts (Corning), and the following day, media was changed to EC medium without basic fibroblast growth factor or retinoic acid. BECs were analyzed for TEER using an EVOM2 instrument (World Precision) to ensure high electrical resistance unique to BECs.

### Bacterial infection

iPSC-derived BECs that are purified onto collagen-fibronectin 24 well plates at 500 k cells/well were grown until Day 10 of the differentiation [23]. A multiplicity of infection (MOI) of 10 was used for all experiments unless specifically noted otherwise as previously described [36]. Overnight cultures of GBS were grown in THB media at 37 °C + 5% CO_2_. The following day bacteria are subcultured and grown to an optical density at 600 nm (OD_600_) of 0.400–0.600. Bacteria are then spun down and washed in phosphate-buffered saline (PBS) prior to infecting BECs. BECs were infected for 5 h at 37 °C at 5% CO_2_ followed by sample collection or assays. Heat killed and formalin-fixed GBS were prepared by growing WT GBS to an OD_600_ of 0.400–0.600 in THB followed by centrifugation and resuspension of the pellet in PBS and treated by heating to 95 °C for 15 min, or treatment of GBS with 4% paraformaldehyde for 15 min. Bacteria were then washed and used to treat BECs at an estimated MOI of 10 for 5 h like live infections described above. Killed GBS preparations were confirmed by plating undiluted fractions onto THB plates where no growth was observed overnight at 37 °C + 5% CO_2_. GBS conditioned media was generated by growing WT GBS in endothelial cell assay media for 5 h, then sterile filtered through a 0.2 μM filter as previously described [19]. Conditioned media preparations were also confirmed sterile by plating undiluted fractions onto THB plates where no growth was observed overnight at 37 °C + 5% CO_2_.

### P-gp activity assay

P-gp activity is assessed by the accumulation of the fluorescent substrates Rhodamine 123 (R123) (Sigma) and FLUO-3-AM (Thermo) [23, 57]. The specific P-gp inhibitor cyclosporine A (CsA) is used as a control to monitor P-gp activity [23]. For other inhibitor experiments PSC-833 (Sigma), MK571 (Sigma), or Ko143 (Sigma) were used to inhibit P-gp, MRPs, and BCRP respectively. Uninfected BECs are used as a control for comparison. After infection, cells are washed with Hank’s Balanced Salt Solution (HBSS) (Thermo) and pre-incubated with or without inhibitor (10 μM CsA, 10 μM PSC-833, 10 μM MK571, or 1 μM Ko143) for 1 h at 37 °C + 5% CO_2_. Following pre-incubation, cells were incubated with 10 μM R123 or FLUO-3-AM with or without inhibitors mentioned for 2 h at 37 °C + 5% CO_2_. After incubation, cells are washed twice in PBS and 200 μl of RIPA buffer (Thermo) is added and placed on a shaker for 10 min at room temperature protected from light. Fluorescence was measured on a plate reader (Tecan). Next, a BCA protein assay (Thermo) was conducted on each well and fluorescence values are normalized to BCA to account for relative cell number as previously described [23].

### Flow cytometry

To assay for the expression of P-gp, BECs were either infected with GBS or left uninfected as a control. After the infection, cells are washed 3× in PBS and 100 μl of Accutase to each well and incubate at 37 °C + 5% CO_2_ to remove cells from the plate. Cells are then fixed by adding 900 μl of 1% paraformaldehyde in PBS for 15 min at room temperature. Cells are then washed twice in wash buffer (5% bovine serum albumin (BSA), 0.1% Triton-X in PBS) to block and permeabilize for the staining of total P-gp. Samples were stained in the wash buffer with anti-P-gp clone F4 (Thermo) (1 μg/million cells) overnight at 4 °C. Following primary stain, samples were washed and stained with secondary anti-mouse 488 (Thermo) at 1:5000 in wash buffer for 1 h at room temperature. Cells were washed and data was collected on a MACSQuant Analyzer 10 (Miltenyi Biotec) and analyzed on FlowJo v10.

### Western blotting

BECs were infected as described above for 5 h at an MOI of 10. After infection, cells were washed three times in PBS and lysates were taken using RIPA buffer (Thermo) plus HALT protease inhibitor cocktail (Thermo). Proteins were quantified using a standard BCA assay kit (Thermo), and equal amounts of protein were loaded onto Nu-PAGE gels (Thermo) and transferred onto nitrocellulose membranes. Membranes were blocked in 5% milk in tris-buffered saline with 0.1% Tween 20 (TBST) and anti-COX-IV antibody 1:1000 (Cell Signaling Technologies) was used as a protein loading control, and anti-P-gp (F4) (Thermo Fisher) was used to determine P-gp abundance. To visualize protein abundance horseradish peroxidase (HRP)-conjugated secondary antibodies (Jackson Laboratory) and a BioRad ChemiDoc XRS + instrument were used to image blots.

### RNA isolation and quantitative PCR

Monolayers of BECs were either left uninfected, or infected with GBS for 5 h at an MOI of 10. After infection, cell lysates and RNA was purified using a NucleoSpin RNA kit (Machery Nagel). cDNA was generated using VILO first-strand synthesis kit (Thermo Fisher), or LunaScript RT (New England BioLabs). SYBR green qPCR was conducted for human *ABCB1* (P-gp, MDR1) forward primer 5′-GAAGAGATTGTGAGGGCAGC-3′, and reverse primer 5′-CCACCAGAGAGCTGAGTTCC-3′. 18S rRNA was used to normalize results and primers used, forward primer 5′-GTAACCCGTTGAACCCCATT-3′ and reverse primer 5′-CCATCCAATCGGTAGTAGCG-3′ were previously described [58]. qPCR data was collected on an Applied Biosystems StepOnePlus and data are presented as fold change using the cycle threshold (ΔΔC_t_) calculation.

### Murine model and staining

Animal experiments were approved by the committee on the use and care of animals at the University of Colorado School of Medicine (protocol #00316) and performed using accepted veterinary standards. The University of Colorado School of Medicine is AAALAC accredited and the facilities meet and adhere to the standards in the “Guide for the Care and Use of Laboratory Animals”. We utilized a mouse GBS infection model as previously described [19, 33, 35]. Briefly, 8 week old male CD-1 mice (Charles River) were injected intravenously with 10^8^–10^9^ of GBS. At the experimental endpoint mice were euthanized and brain tissue was collected. One half of the brain was frozen in OCT compound (Sakura) and sectioned using a CM1950 cryostat (Leica). Sections were fixed with ice cold methanol (Sigma) for 20 min, blocked with Mouse on Mouse blocking reagent (Vector Labs), and incubated with mouse anti P-glycoprotein antibody clone C219 (ThermoFisher) overnight at 4 °C followed by goat anti-mouse conjugated to Cy3 (Jackson Immunoresearch) and tomato lectin conjugated to DyLight488 (Vector Labs). Coverslips were mounted with Fluoroshield + DAPI (Abcam) and images were taken using a BZ-X710 microscope (Keyence). Pixel intensities of P-gp staining were estimated by using the FIJI image analysis program. Briefly, lectin positive vessels were traced manually and the selection was transposed onto the P-gp image and mean pixel intensity was measured.

### Immunofluorescence

iPSC-BECs were fixed and stained for markers exactly as described previously [23], with the exceptions that anti-CD-31 (Abcam, cat# ab32457), and anti-ZO1 (Proteintech, cat# 21773-1-AP) were utilized in place of referenced reagents. Briefly, BECs were fixed for 15 min in cold methanol and stained overnight at 4 °C. The following day, secondary antibodies anti-mouse 488 (Invitrogen, cat# A11001), and anti-rabbit 555 (Invitrogen, cat# A31572) were used at a dilution of 1:200. Samples were then visualized on a Nikon Eclipse T*i* using Nikon NIS image acquisition software.

### Statistics

GraphPad Prism version 5.0 (GraphPad Software Inc.) was used for all statistical analysis. For pairwise comparison, 2-tailed Student’s *t* test was used where appropriate. For multiple comparisons, analysis of variance (ANOVA) followed by Dunnett’s multiple comparisons test was used where appropriate. Data are represented as mean, ± standard deviation (SD) since all raw values are presented. Statistical significance was accepted at a *P* value of less than 0.05.

## Additional files

**Additional file 1: Figure S1.** Characterization of iPSC-BECs. (A-F) Representative immunofluorescence images of differentiated iPSC-BECs. (A) VE-cadherin, (B) CD-31, (C) Claudin-5, (D) Occludin, (E) ZO-1, (F) Glut-1. Images were taken using a 20× objective and scale bar represents 50 μm.

**Additional file 2: Figure S2.** Additional staining of different mice for lectin (green) and P-gp (red), and DAPI (blue). Two representative images from two control mice (top). Two representative images from two GBS mice (bottom). Scale bar represents 50 μm.

## Data Availability

The datasets used and/or analyzed during the current study are available from the corresponding author on reasonable request. Specifically regarding the representative western blots, flow cytometry histogram, and brain images from mice (Fig. 3). All other data sets generated or analyzed during this study are included in this published article.

## Abbreviations

BECs: brain endothelial cells
CNS: central nervous system
P-gp: *P*-glycoprotein
iPSC: induced pluripotent stem cell
CsA: cyclosporine A
GBS: group B *Streptococcus*
MOI: multiplicity of infection
R123: rhodamine 123
WT: wild-type
MDCK: Madin–Darby Canine Kidney
qPCR: quantitative polymerase chain reaction
OCT: optimal cutting temperature
CFU: colony forming unit
LPS: lipopolysaccharide
FOV: field of view
BCRP: breast cancer resistance protein
MRP: multi-drug resistance protein
