# Supplementary figures and images for: Streptococcus agalactiae disrupts P-glycoprotein function in brain endothelial cells
Source: Fluids Barriers CNS. 2019 Aug 22;16:26. doi: 10.1186/s12987-019-0146-5 (PMC6704684; doi:10.1186/s12987-019-0146-5)

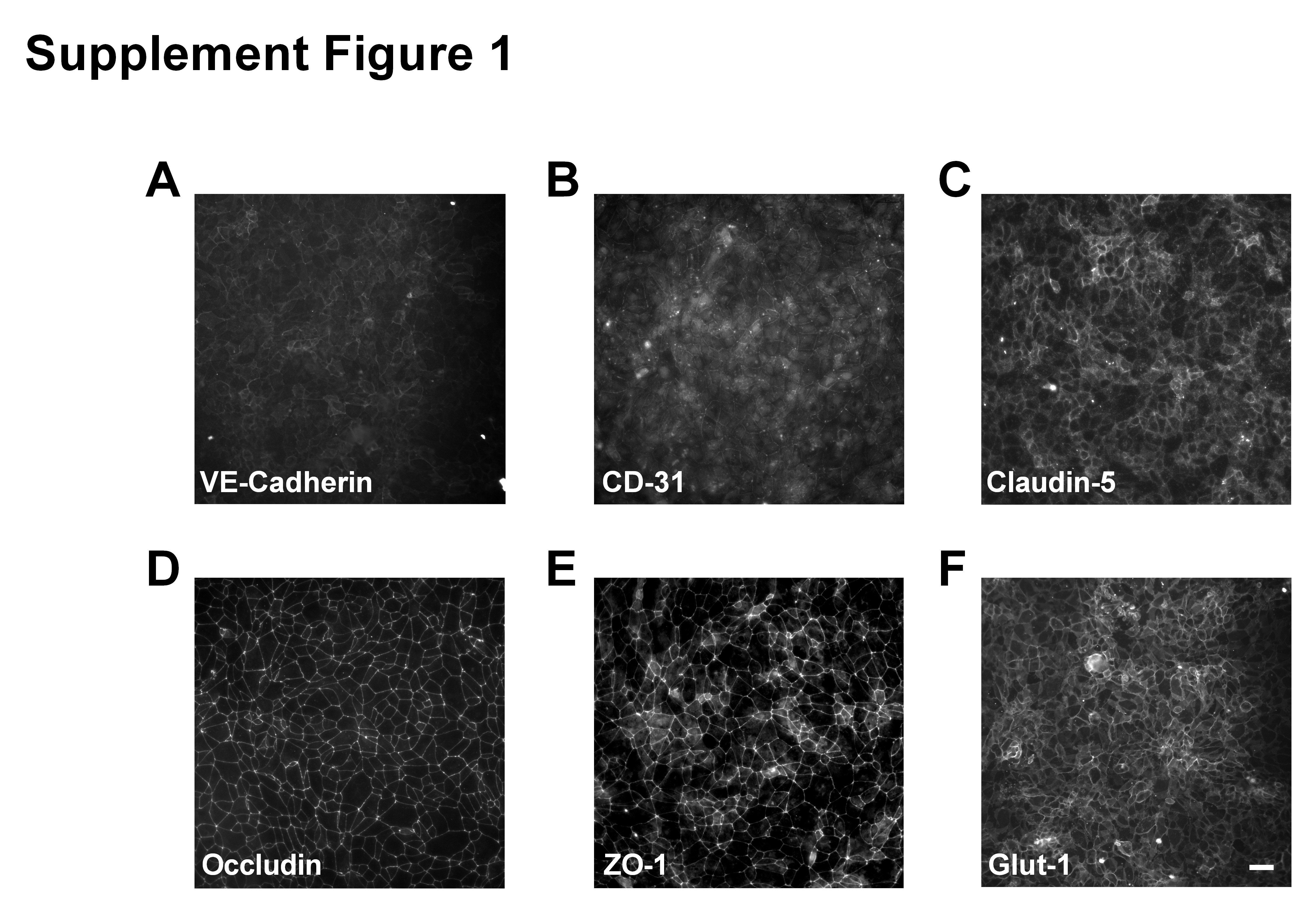

Supplement: Supplementary file 1 — Additional file 1: Figure S1. Characterization of iPSC-BECs. (A-F) Representative immunofluorescence images of differentiated iPSC-BECs. (A) VE-cadherin, (B) CD-31, (C) Claudin-5, (D) Occludin, (E) ZO-1, (F) Glut-1. Images were taken using a 20× objective and scale bar represents 50 μm. [file 12987_2019_146_MOESM1_ESM.jpg]

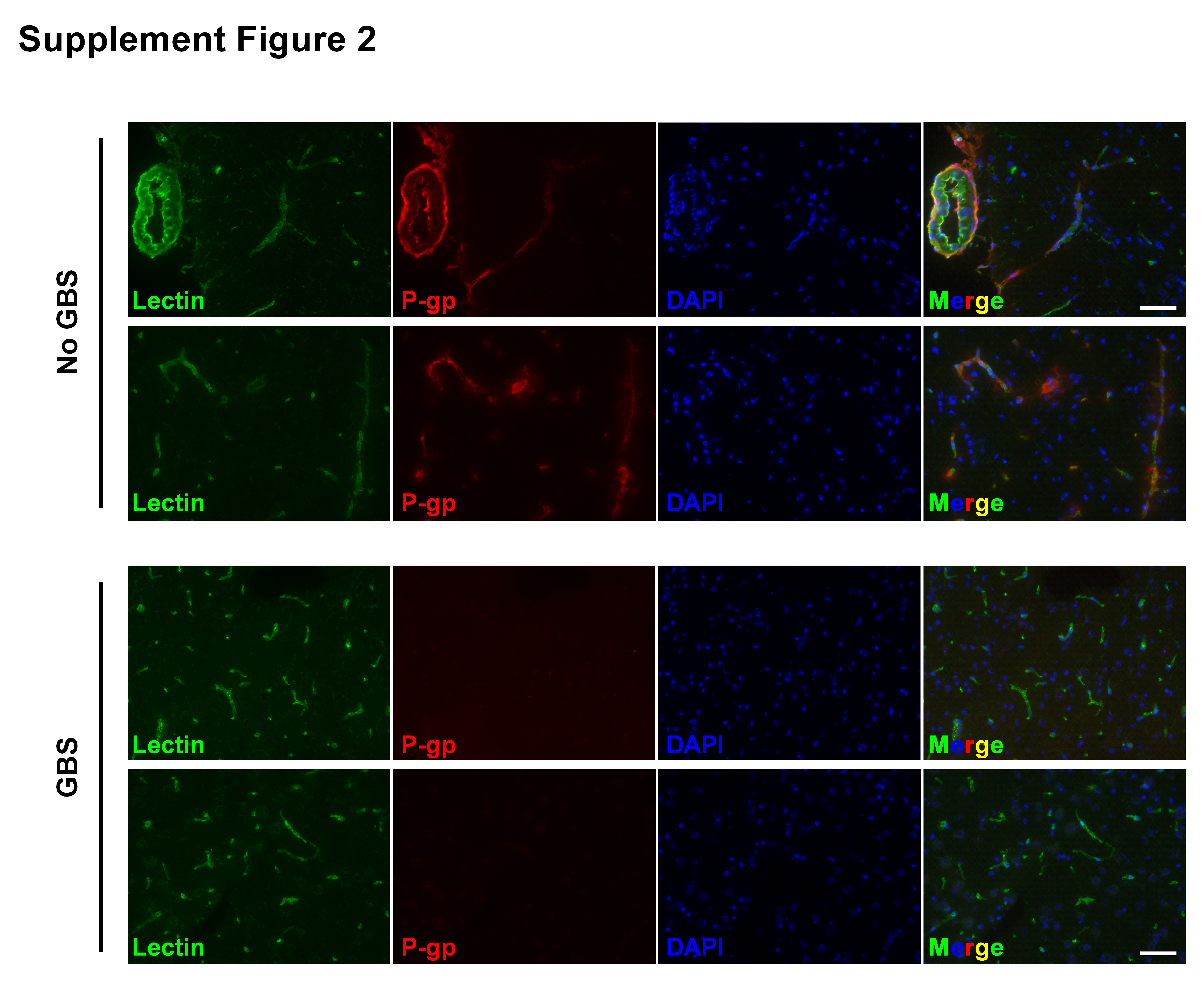

Supplement: Supplementary file 2 — Additional file 2: Figure S2. Additional staining of different mice for lectin (green) and P-gp (red), and DAPI (blue). Two representative images from two control mice (top). Two representative images from two GBS mice (bottom). Scale bar represents 50 μm. [file 12987_2019_146_MOESM2_ESM.jpg]
